# Supplementary material for: The Systems Biology Research Tool: evolvable open-source software
Source: BMC Syst Biol. 2008 Jun 29;2:55. doi: 10.1186/1752-0509-2-55 (PMC2446383; doi:10.1186/1752-0509-2-55)
Supplement: Additional file 1 — SBRT Archive. An archive of the current version of the Systems Biology Research Tool. [file 1752-0509-2-55-S1.zip › sbrt-1.4.0/doc/developers_guide/api/sbrt/shell/mng/AbstractProcessManager.html]

AbstractProcessManager


|  |  |  |
| --- | --- | --- |
| **Overview** \| **Package** \| **Class** \| **Use** \| **Tree** \| **Deprecated** \| **Index** \| **Help** | |  |
| PREV CLASS   **NEXT CLASS** | **FRAMES**    **NO FRAMES**     **All Classes** |  |
| SUMMARY: NESTED \| FIELD \| CONSTR \| METHOD | DETAIL: FIELD \| CONSTR \| METHOD |  |


---


## sbrt.shell.mng Class AbstractProcessManager

```
java.lang.Object
  sbrt.shell.mng.AbstractProcessManager
```

**All Implemented Interfaces:**: ProcessManager<java.lang.String,java.lang.String>

**Direct Known Subclasses:**: BiggSbmlReaderManager, BiggSbmlTranslaterManager, CdHarManager, CorrelationManager, CycleIdManager, FbaManager, IntervalComparisonManager, KendallTauManager, LinearCombConverterManager, LinearSolverManager, MannWhitneyUManager, MatrixConverterManager, MetatoolFileWriterManager, MmaNotebookManager, MultipleVectorsConverterManager, PalssonSbmlReaderManager, PalssonSbmlTranslaterManager, PathIdManager, RxnRemovalManager, SbmlWriterManager, SimpleRxnFileTransManager, SingleElementUnionManager, SingleVectorConverterManager, StrictSingleElementUnionManager, UniqueCycleIdManager, VariableParticipationManager, VectorComparisonManager

---

``` public abstract class AbstractProcessManager extends java.lang.Object implements ProcessManager<java.lang.String,java.lang.String> ```

This class is a skeleton implemenation of `KernelProcessManager`.

**Author:**
:   This class was written and documented by Jeremiah Wright while in the
    Wagner lab.

---

| **Field Summary** | |
| --- | --- |
| `static java.lang.String` | `ALGORITHM`             The keyword used to denote an algorithm. |
| `static java.lang.String` | `COMPLETED`             The prefix used to denote the number of completed operations. |
| `static java.lang.String` | `DATA_HEADERS`             The keyword used to denote the data headers of, say, an output file. |
| `static java.io.PrintWriter` | `DEV_NULL`             A print writer that discards all data it receives. |
| `static java.lang.String` | `ELAPSED_TIME`             The prefix used to denote the amount of time that has elapsed since an operation was started. |
| `static java.lang.String` | `FILE_FORMAT`             The keyword used to denote the name of a file format. |
| `static java.lang.String` | `INPUT_FILE`             The keyword used to denote the name of the input file. |
| `static java.lang.String` | `INPUT_FILE_FORMAT`             The keyword used to denote the name of an input file format. |
| `static java.lang.String` | `INPUT_FILE_NAME_FILE`             The keyword used to denote the name of a file containing a list of file names. |
| `static java.lang.String` | `ITERATIONS`             The keyword used to denote the number of iterations. |
| `static UnmodifiableSet<java.lang.String>` | `optionalKeywords`             The set of optional keywords in the `ManagerInput` objects supplied to instances of this class. |
| `static java.lang.String` | `OUTPUT_FILE_FORMAT`             The keyword used to denote the name of an output file format. |
| `static java.lang.String` | `OUTPUT_FILE_NAME`             The keyword used to denote the name of the output file. |
| `static java.lang.String` | `OUTPUT_FILE_NAME_FILE`             The keyword used to denote the name of a file containing a list of file names for other output files. |
| `static PercentFormat` | `PERCENT`             The default format used for percentages. |
| `static java.lang.String` | `PERCENT_COMPLETED`             The prefix used to denote the completed percentage of an operation. |
| `static UnmodifiableSet<java.lang.String>` | `requiredKeywords`             The set of keywords required to be present in the `ManagerInput` objects supplied to instances of this class. |
| `static java.lang.String` | `SEED`             The keword used to denote the seed to a pseudorandom number generator. |
| `static java.lang.String` | `ZERO_CUTOFF`             The keyword used to denote the maximum numerical value that will still be considered equal to zero. |


**Constructor Summary**

`AbstractProcessManager()`


| **Method Summary** | |
| --- | --- |
| `void` | `check(ManagerInput<java.lang.String,java.lang.String> input)`             Throws an ApplicationException if this `ProcessManager` cannot understand the provided input. |
| `static void` | `closeFile(SbrtFile file, java.io.PrintWriter out)`             Closes the provided results manager and prints a descriptive message to the provided print writer. |
| `static void` | `closeRm(ResultsManager rm, java.io.PrintWriter out)`             Closes the provided file and prints a descriptive message to the provided print writer. |
| `void` | `execute()`             Executes the process this process manager controls. |
| `static FileFormat` | `getFileFormat(ManagerInput<java.lang.String,java.lang.String> input)`             Returns the parsed value for `FILE_FORMAT` in the provided input. |
| `static FileFormat` | `getFileFormat(java.lang.String formatName)`             Parses and returns the file format corresponding to the provided string. |
| `static TextInputFile<java.lang.String>` | `getFileNameSource(ManagerInput<java.lang.String,java.lang.String> input, java.io.PrintWriter out)`             Returns the parsed value for `OUTPUT_FILE_NAME_FILE` in the provided input and prints a descriptive message to the provided print writer. |
| `static int` | `getIterations(ManagerInput<java.lang.String,java.lang.String> input, java.io.PrintWriter out)`             Returns the parsed value for `ITERATIONS` in the provided input and prints a descriptive message to the provided print writer. |
| `static long` | `getSeed(ManagerInput<java.lang.String,java.lang.String> input, java.io.PrintWriter out)`             Returns the parsed value for `SEED` in the provided input and prints a descriptive message to the provided print writer. |
| `static FileFormat` | `getTextFileFormat(ManagerInput<java.lang.String,java.lang.String> input)`             Returns the parsed value for `FILE_FORMAT` in the provided input. |
| `static FileFormat` | `getTextFileFormat(java.lang.String fileFormatString)`             Returns the text file format corresponding to the provided string. |
| `static double` | `getZeroCutoff(ManagerInput<java.lang.String,java.lang.String> input)`             Returns the parsed value for `ZERO_CUTOFF` in the provided input. |
| `static double` | `getZeroCutoff(ManagerInput<java.lang.String,java.lang.String> input, java.io.PrintWriter out)`             Returns the parsed value for `ZERO_CUTOFF` in the provided input. |
| `static int` | `parseInt(java.lang.String number)`             Parses the provided string as a `int`. |
| `static long` | `parseLong(java.lang.String number)`             Parses the provided string as a `long`. |
| `static void` | `printProgress(java.lang.Double progress, java.io.PrintWriter out)`             Prints the provided progress to the provided print writer. |
| `static void` | `printProgress(java.lang.Integer progress, java.io.PrintWriter out)`             Prints the provided progress to the provided print writer. |
| `static void` | `printProgress(java.lang.Number progress, java.io.PrintWriter out)`             Prints the provided progress to the provided print writer. |
| `static <T> void` | `runProcess(MonitoredProcess<T> process, ResultsManager<T> resultsManager, java.io.PrintWriter out)`             Runs the provided process using the provided results manager and prints descriptive progress messages to the provided print writer. |
| `void` | `setInput(ManagerInput<java.lang.String,java.lang.String> input)`             Uses the provided input to prepare this process manager for execution. |

**Methods inherited from class java.lang.Object**

`clone, equals, finalize, getClass, hashCode, notify, notifyAll, toString, wait, wait, wait`

**Methods inherited from interface sbrt.shell.mng.ProcessManager**

`execute, getOptionalKeys, getRequiredKeys, setInput`

**Field Detail**

### DEV\_NULL

```
public static final java.io.PrintWriter DEV_NULL
```

:   A print writer that discards all data it receives.

---


### PERCENT

```
public static PercentFormat PERCENT
```

:   The default format used for percentages.

---


### ZERO\_CUTOFF

```
public static final java.lang.String ZERO_CUTOFF
```

:   The keyword used to denote the maximum numerical value that will still be
    considered equal to zero.

    **See Also:**: Constant Field Values

---


### SEED

```
public static final java.lang.String SEED
```

:   The keword used to denote the seed to a pseudorandom number generator.

    **See Also:**: Constant Field Values

---


### COMPLETED

```
public static final java.lang.String COMPLETED
```

:   The prefix used to denote the number of completed operations.

    **See Also:**: Constant Field Values

---


### PERCENT\_COMPLETED

```
public static final java.lang.String PERCENT_COMPLETED
```

:   The prefix used to denote the completed percentage of an operation.

    **See Also:**: Constant Field Values

---


### ELAPSED\_TIME

```
public static final java.lang.String ELAPSED_TIME
```

:   The prefix used to denote the amount of time that has elapsed since an
    operation was started.

    **See Also:**: Constant Field Values

---


### ALGORITHM

```
public static final java.lang.String ALGORITHM
```

:   The keyword used to denote an algorithm.

    **See Also:**: Constant Field Values

---


### ITERATIONS

```
public static final java.lang.String ITERATIONS
```

:   The keyword used to denote the number of iterations.

    **See Also:**: Constant Field Values

---


### OUTPUT\_FILE\_NAME

```
public static final java.lang.String OUTPUT_FILE_NAME
```

:   The keyword used to denote the name of the output file.

    **See Also:**: Constant Field Values

---


### INPUT\_FILE\_NAME\_FILE

```
public static final java.lang.String INPUT_FILE_NAME_FILE
```

:   The keyword used to denote the name of a file containing a list of file
    names.

    **See Also:**: Constant Field Values

---


### OUTPUT\_FILE\_NAME\_FILE

```
public static final java.lang.String OUTPUT_FILE_NAME_FILE
```

:   The keyword used to denote the name of a file containing a list of file
    names for other output files.

    **See Also:**: Constant Field Values

---


### DATA\_HEADERS

```
public static final java.lang.String DATA_HEADERS
```

:   The keyword used to denote the data headers of, say, an output file.

    **See Also:**: Constant Field Values

---


### INPUT\_FILE

```
public static final java.lang.String INPUT_FILE
```

:   The keyword used to denote the name of the input file.

    **See Also:**: Constant Field Values

---


### FILE\_FORMAT

```
public static final java.lang.String FILE_FORMAT
```

:   The keyword used to denote the name of a file format.

    **See Also:**: Constant Field Values

---


### OUTPUT\_FILE\_FORMAT

```
public static final java.lang.String OUTPUT_FILE_FORMAT
```

:   The keyword used to denote the name of an output file format.

    **See Also:**: Constant Field Values

---


### INPUT\_FILE\_FORMAT

```
public static final java.lang.String INPUT_FILE_FORMAT
```

:   The keyword used to denote the name of an input file format.

    **See Also:**: Constant Field Values

---


### requiredKeywords

```
public static final UnmodifiableSet<java.lang.String> requiredKeywords
```

:   The set of keywords required to be present in the `ManagerInput`
    objects supplied to instances of this class.

---


### optionalKeywords

```
public static final UnmodifiableSet<java.lang.String> optionalKeywords
```

:   The set of optional keywords in the `ManagerInput` objects supplied
    to instances of this class.


**Constructor Detail**

### AbstractProcessManager

```
public AbstractProcessManager()
```


**Method Detail**

### getFileNameSource

```
public static TextInputFile<java.lang.String> getFileNameSource(ManagerInput<java.lang.String,java.lang.String> input,
                                                                java.io.PrintWriter out)
```

:   Returns the parsed value for `OUTPUT_FILE_NAME_FILE` in the
    provided input and prints a descriptive message to the provided print
    writer.

    :   **Parameters:**: `input` - the input whose `OUTPUT_FILE_NAME_FILE` value is to be parsed.: `out` - the print writer to which a message is printed. **Returns:**: a text-based input file containing the names of output files.

---


### check

```
public void check(ManagerInput<java.lang.String,java.lang.String> input)
```

:   Throws an ApplicationException if this `ProcessManager` cannot
    understand the provided input.

    :   **Specified by:**: `check` in interface `ProcessManager<java.lang.String,java.lang.String>`
    :   **Parameters:**: `input` - ManagerInput

---


### runProcess

```
public static <T> void runProcess(MonitoredProcess<T> process,
                                  ResultsManager<T> resultsManager,
                                  java.io.PrintWriter out)
```

:   Runs the provided process using the provided results manager and prints
    descriptive progress messages to the provided print writer.

    :   **Type Parameters:**: `T` - the results type. **Parameters:**: `process` - the process to run.: `resultsManager` - the results manager with which to supply the process.: `out` - the print writer where descriptive progress messages will be written.

---


### closeRm

```
public static void closeRm(ResultsManager rm,
                           java.io.PrintWriter out)
```

:   Closes the provided file and prints a descriptive message to the provided
    print writer. If the file is already closed, nothing is done.

    :   **Parameters:**: `rm` - the results manager to close.: `out` - the print writer to which a message will be printed. **Throws:**: `java.lang.NullPointerException` - if either argument is null.

---


### closeFile

```
public static void closeFile(SbrtFile file,
                             java.io.PrintWriter out)
```

:   Closes the provided results manager and prints a descriptive message to
    the provided print writer. If the file is already closed, nothing is
    done.

    :   **Parameters:**: `file` - the file to close.: `out` - the print writer to which a message will be printed. **Throws:**: `java.lang.NullPointerException` - if either argument is null.

---


### printProgress

```
public static void printProgress(java.lang.Number progress,
                                 java.io.PrintWriter out)
```

:   Prints the provided progress to the provided print writer.

    :   **Parameters:**: `progress` - a number representing a progress.: `out` - the print writer to which the progress will be printed. **Throws:**: `java.lang.NullPointerException` - if either argument is null.

---


### printProgress

```
public static void printProgress(java.lang.Integer progress,
                                 java.io.PrintWriter out)
```

:   Prints the provided progress to the provided print writer.

    :   **Parameters:**: `progress` - a number representing a progress.: `out` - the print writer to which the progress will be printed. **Throws:**: `java.lang.NullPointerException` - if either argument is null.

---


### printProgress

```
public static void printProgress(java.lang.Double progress,
                                 java.io.PrintWriter out)
```

:   Prints the provided progress to the provided print writer.

    :   **Parameters:**: `progress` - a number representing a progress.: `out` - the print writer to which the progress will be printed. **Throws:**: `java.lang.NullPointerException` - if either argument is null.

---


### parseLong

```
public static long parseLong(java.lang.String number)
```

:   Parses the provided string as a `long`.

    :   **Parameters:**: `number` - the string to parse. **Returns:**: the `long` value represented by the provided string.

---


### parseInt

```
public static int parseInt(java.lang.String number)
```

:   Parses the provided string as a `int`.

    :   **Parameters:**: `number` - the string to parse. **Returns:**: the `int` value represented by the provided string.

---


### getSeed

```
public static long getSeed(ManagerInput<java.lang.String,java.lang.String> input,
                           java.io.PrintWriter out)
```

:   Returns the parsed value for `SEED` in the provided input and
    prints a descriptive message to the provided print writer. If the
    provided input does not contain a value for `SEED`, the current
    time in milliseconds is returned instead.

    :   **Parameters:**: `input` - the input whose `SEED` value is to be parsed.: `out` - the print writer to which a message is printed. **Returns:**: the parsed seed value; or System.currentTimeMillis() if no value is present.

---


### getIterations

```
public static int getIterations(ManagerInput<java.lang.String,java.lang.String> input,
                                java.io.PrintWriter out)
```

:   Returns the parsed value for `ITERATIONS` in the provided input
    and prints a descriptive message to the provided print writer.

    :   **Parameters:**: `input` - the input whose `ITERATIONS` value is to be parsed.: `out` - the print writer to which a message is printed. **Returns:**: the parsed number of iterations. **Throws:**: `ApplicationException` - if the parsed integer is less than 0.

---


### getFileFormat

```
public static FileFormat getFileFormat(ManagerInput<java.lang.String,java.lang.String> input)
```

:   Returns the parsed value for `FILE_FORMAT` in the provided input.

    :   **Parameters:**: `input` - the input whose `FILE_FORMAT` value is to be parsed. **Returns:**: the parsed file format.

---


### getTextFileFormat

```
public static FileFormat getTextFileFormat(java.lang.String fileFormatString)
```

:   Returns the text file format corresponding to the provided string.

    :   **Parameters:**: `fileFormatString` - a string parsable by FileFormatFormat. **Returns:**: FileFormat.TEXT if the provided string is `null`, or FileFormat.TEXT or FileFormat.GZIP\_TEXT. **Throws:**: `ApplicationException` - if the provided string cannot be parsed, or is parsed as any file format other than plain or gzipped text.

---


### getTextFileFormat

```
public static FileFormat getTextFileFormat(ManagerInput<java.lang.String,java.lang.String> input)
```

:   Returns the parsed value for `FILE_FORMAT` in the provided input.

    :   **Parameters:**: `input` - the input whose `FILE_FORMAT` value is to be parsed. **Returns:**: the parsed file format. **Throws:**: `ApplicationException` - if the parsed format is not a valid text-based file format.

---


### getFileFormat

```
public static FileFormat getFileFormat(java.lang.String formatName)
```

:   Parses and returns the file format corresponding to the provided string.

    :   **Parameters:**: `formatName` - the name of a file format. **Returns:**: the parsed file format.

---


### getZeroCutoff

```
public static double getZeroCutoff(ManagerInput<java.lang.String,java.lang.String> input,
                                   java.io.PrintWriter out)
```

:   Returns the parsed value for `ZERO_CUTOFF` in the provided input.

    :   **Parameters:**: `input` - the input whose `ZERO_CUTOFF` value is to be parsed.: `out` - the print writer to which a status message will be printed. **Returns:**: the parsed numerical zero cutoff; or 0.0d if no value for `ZERO_CUTOFF` exists. **Throws:**: `ApplicationException` - if the parsed cutoff is infinite, NaN, or < 0.

---


### getZeroCutoff

```
public static double getZeroCutoff(ManagerInput<java.lang.String,java.lang.String> input)
```

:   Returns the parsed value for `ZERO_CUTOFF` in the provided input.

    :   **Parameters:**: `input` - the input whose `ZERO_CUTOFF` value is to be parsed. **Returns:**: the parsed numerical zero cutoff. **Throws:**: `ApplicationException` - if the parsed cutoff is infinite, NaN, or < 0.

---


### setInput

```
public void setInput(ManagerInput<java.lang.String,java.lang.String> input)
```

:   Uses the provided input to prepare this process manager for execution.

    :   **Specified by:**: `setInput` in interface `ProcessManager<java.lang.String,java.lang.String>`
    :   **Parameters:**: `input` - all of the information required to prepare this process manager for execution.

---


### execute

```
public void execute()
```

:   Executes the process this process manager controls.

    :   **Specified by:**: `execute` in interface `ProcessManager<java.lang.String,java.lang.String>`
    :   **Throws:**: `java.lang.IllegalStateException` - if no input was provided to this process manager.


---


|  |  |  |
| --- | --- | --- |
| **Overview** \| **Package** \| **Class** \| **Use** \| **Tree** \| **Deprecated** \| **Index** \| **Help** | |  |
| PREV CLASS   **NEXT CLASS** | **FRAMES**    **NO FRAMES**     **All Classes** |  |
| SUMMARY: NESTED \| FIELD \| CONSTR \| METHOD | DETAIL: FIELD \| CONSTR \| METHOD |  |


---
